# Acceptance and attitudes of healthcare staff towards the introduction of clinical pharmacy service: a descriptive cross-sectional study from a tertiary care hospital in Sri Lanka

**DOI:** 10.1186/s12913-017-2001-1

**Published:** 2017-01-18

**Authors:** Lelwala Guruge Thushani Shanika, Chandrani Nirmala Wijekoon, Shaluka Jayamanne, Judith Coombes, Ian Coombes, Nilani Mamunuwa, Andrew Hamilton Dawson, Hithanadura Asita De Silva

**Affiliations:** 1 Department of Allied Health Sciences, Faculty of Medical Sciences, University of Sri Jayewardenepura, Sri Jayewardenepura Kotte, Sri Lanka; 2 South Asian Clinical Toxicology Research Collaboration, Peradeniya, Sri Lanka; 3 Department of Pharmacology, Faculty of Medical Sciences, University of Sri Jayewardenepura, Sri Jayewardenepura Kotte, Sri Lanka; 4 Department of Medicine, Faculty of Medicine, University of Kelaniya, Kelaniya, Sri Lanka; 5 Princess Alexandra Hospital, Brisbane, Australia; 6 University of Queensland, Brisbane, Australia; 7 Royal Brisbane Hospital, Brisbane, Australia; 8 Central Clinical School, Faculty of Medicine, University of Sydney, Sydney, Australia; 9 Clinical Trials Unit, Department of Pharmacology, Faculty of Medicine, University of Kelaniya, Kelaniya, Sri Lanka

**Keywords:** Clinical pharmacist, Acceptance, Attitudes, Doctors, Nurses, Multidisciplinary team, Drug- related problems

## Abstract

**Background:**

Multidisciplinary patient management including a clinical pharmacist shows an improvement in patient quality use of medicine. Implementation of a clinical pharmacy service represents a significant novel change in practice in Sri Lanka. Although attitudes of doctors and nurses are an important determinant of successful implementation, there is no Sri Lankan data about staff attitudes to such changes in clinical practice. This study determines the level of acceptance and attitudes of doctors and nurses towards the introduction of a ward-based clinical pharmacy service in Sri Lanka.

**Methods:**

This is a descriptive cross-sectional sub-study which determines the acceptance and attitudes of healthcare staff about the introduction of a clinical pharmacy service to a tertiary care hospital in Sri Lanka. The level of acceptance of pharmacist’s recommendations regarding drug-related problems (DRPs) was measured. Data regarding attitudes were collected through a pre-tested self-administered questionnaires distributed to doctors (baseline, *N* =13, post-intervention period, *N* = 12) and nurses (12) worked in professorial medical unit at baseline and post-intervention period.

**Results:**

A total of 274 (272 to doctors and 2 to nurses) recommendations regarding DRPs were made. Eighty three percent (225/272) and 100% (2/2) of the recommendations were accepted by doctors and nurses, respectively. The rate of implementation of pharmacist’s recommendations by doctors was 73.5% (200/272) (95% CI 67.9 – 78.7%; *P* < 0.001). The response rate of doctors was higher at the post-intervention period (92.3%; 12/13) compared to the baseline (66.7%; 8/12). At the post-intervention survey 91.6% of doctors were happy to work with competent clinical pharmacists and accepted the necessity of this service to improve standards of care. The nurses’ rate of response at baseline and post-intervention surveys were 80.0 and 0.0% respectively. Their perceptions on the role of clinical pharmacist were negative at baseline survey.

**Conclusions:**

There was high acceptance and implementation of clinical pharmacist’s recommendations regarding DRPs by the healthcare team. The doctors’ views and attitudes were positive regarding the inclusion of a ward-based pharmacist to the healthcare team. However there is a need to improve liaison between clinical pharmacist and nursing staff.

**Trial registration:**

Sri Lanka Clinical Trials Registry SLCTR/2013/029 Date: 13 September 2013; retrospectively registered.

**Electronic supplementary material:**

The online version of this article (doi:10.1186/s12913-017-2001-1) contains supplementary material, which is available to authorized users.

## Background

Medication use is one of the most common types of interventions in healthcare and it is ubiquitous. Many years ago, most Western and European countries identified the positive impact of a clinical pharmacy service in improving quality use of medicines (QUM) which in turn improves overall patient care [1].

However, collaboration between medical staff and clinical pharmacists in providing patient care is a novel concept in Sri Lanka. At present pharmacists are not a part of the multidisciplinary healthcare team and their role is limited to drug dispensing, providing limited medicine information and compounding within the hospital.

Non-communicable chronic diseases (NCCDs) comprise a major group of diseases that necessitate the long term use of medications. NCCDs are a significant global health challenge affecting both the developed and developing world [2, 3]. Sri Lanka is a developing country with a high burden of NCCDs that in 2012 accounted for almost one-third of hospital admissions [4].

In many countries hospital treatment of NCCDs utilizes multidisciplinary teams that include clinical pharmacy services [5–7]. McAlister, et al. in their systematic review of randomized controlled trials had revealed that multidisciplinary approaches that included clinical pharmacy services in managing patients with heart failure had reduced mortality and morbidity [6]. Another systematic review analyzing 126 studies in United States showed a cost benefit on health outcomes in pharmacist directed pharmaceutical care services [8].

In order to inform policy makers in Sri Lanka regarding future developments in health care delivery we conducted a study to evaluate the implementation, impact, and acceptability of the introduction of a ward-based clinical pharmacy service as a novel intervention in a tertiary care hospital in Sri Lanka [9, 10].

This paper describes the level of acceptance of the clinical pharmacy service; in the aforementioned primary study, by the other members of the healthcare team. This sub-study utilizes the observational data gathered from the intervention arm of the clinical trial and data gathered from a separate staff survey. The aims of this sub-study were to determine the level of acceptance of the clinical pharmacist’s recommendations to resolve Drug Related Problems (DRPs) by the healthcare staff, to determine the quantity and quality of drug information queries directed to the clinical pharmacist from other staff and to assess the views and attitudes of the other members of healthcare staff towards the clinical pharmacy service.

## Methods

### Study design, setting and procedure

We established a controlled clinical trial to assess the effectiveness of a clinical pharmacy service in improving QUM in patients with NCCDs. This paper described a descriptive cross-sectional sub-study of abovementioned controlled trial under the area of the acceptance and attitudes of healthcare staff on the introduction of a clinical pharmacy service to a tertiary care hospital in Sri Lanka. Within the intervention arm acceptance of the clinical pharmacy service was measured by quantitatively examining the rate of acceptance of the clinical pharmacist’s recommendations regarding DRPs by the healthcare staff and by quantitatively and qualitatively examining the drug information queries directed to the clinical pharmacist by the healthcare staff. Staff attitudes towards the acceptance of the clinical pharmacy service was determined by repeated surveys which conducted before and after the trial intervention period.

#### Clinical trial

The clinical trial was conducted over a thirteen months period (recruitment from March 2013 to September 2013; follow-up from October 2013 to March 2014), in the University Medical Unit of Colombo North Teaching Hospital, Sri Lanka; a large tertiary care teaching hospital having 1421 beds with more than 500,000 admissions annually. There was no established ward-based pharmacy service in the hospital and the existing hospital pharmacy service was limited to dispensing and it did not have a formal process for pharmacists’ provision of medication education to patients. The University Medical Unit consists a female and a male ward accommodating approximately 55 and 65 patients, respectively.

#### Study population

Eligible patients were those with non-communicable chronic diseases (NCCDs) who needed long-term follow-up at the medical clinic. Each day the first five eligible patients in each ward as recorded chronologically in the admission register were approached by an independent medical officer to be consented and recruited to the study.

In the first 3 months of the study, patients from the male ward received the intervention and patients from the female ward were recruited to the control arm. In the second 3 months of the study, patients from the female ward received the intervention and patients from the male ward were recruited to the control arm. The control group received usual care which did not include clinical pharmacist’s input whereas the intervention group received a clinical pharmacy service in addition to usual management.

##### Clinical pharmacist’s interventions

The intervention group patients were interviewed for medication history on the day of admission and followed up prospectively during their hospital stay by the clinical pharmacist. The pharmacist did medication reconciliation; compared the medications the patient was on prior to admission with the medications prescribed on the drug chart after admission and discrepancies were communicated to the medical team. During the hospital stay, the pharmacist did a prospective medication review daily and all the clinically important DRPs were also discussed with the healthcare team (doctors and nurses) and appropriate recommendations made. The pharmacist also educated patients regarding their medications and provided medication information to the healthcare team when needed. The pharmacist took part in the ward rounds weekly to support multidisciplinary patient management.

##### Training received by clinical pharmacist

The intervention pharmacist was a B. Pharm graduate and clinical pharmacy was a subject in her undergraduate curriculum. Before and during the study period the pharmacists received ward-based teaching from Australian clinical pharmacy educators and weekly Skype™ case based conference tutorials were also undertaken with senior clinical pharmacists from Australia.

#### Staff survey

The views and general perspectives of healthcare staff was assessed through a self-administered questionnaire at baseline (before introducing the clinical pharmacy service) and at the end of the trial (post-intervention) to determine any change in the views and attitudes of doctors and nurses towards the clinical pharmacy service.

This questionnaire was developed and pre-tested in a pilot study and reviewed by a team of clinical pharmacists and consultant physicians prior to the study. The questionnaire was developed in such a way that it sought the general perspectives of health staff on how this integrated medicine management related to both patients and to the healthcare staff. In the questionnaire there were 17 questions in total. The answers to the first 9 close-ended questions were designed using a five-grade Likert scale, ranging from “strongly agree”, “agree”, “no opinion”, “disagree” and “strongly disagree” (Additional file 1: Table S1 and Additional file 2: Table S3). The other questions were customized for medical or nursing staff and addressed specific tasks of the clinical pharmacy service provided (Additional file 3: Table S2 and Additional file 4: Table S4). These questions were answered with a dichotomized response alternative, “Yes” or “No”. An independent research assistant distributed the questionnaires among all the healthcare members (consultant physicians, senior-registrars, registrars, grade medical officers, intern house officers and nursing staff) who worked in the University Medical Unit during the relevant study period were included and the completed questionnaires were collected in a sealed box kept in the staff room. Participants were given a participant information sheet and in addition they were informed about the purpose of the survey verbally by the investigator. An individual consent form was attached with the questionnaire and it clearly explained the purpose of the survey and the capability of participants to voluntarily withhold their consent.

### Study outcomes

The study outcomes included level of acceptance of the pharmacist’s recommendations regarding DRPs, quantity and nature of drug information queries directed to the clinical pharmacist by the healthcare staff and the views and attitudes of the healthcare staff towards clinical pharmacy service.

The clinical pharmacist identified and classified DRPs according to the adapted Pharmaceutical Care Network Europe (PCNE) classification system V5.01 [11]. A subset of 52% of DRPs were externally validated by Australian clinical pharmacy team and two consultant physicians involved in the study. The pharmacist’s recommendations were identified as “Accepted” (the healthcare staff accepted the pharmacist’s recommendations) and “Not accepted” (the staff did not agree with the recommendations) interventions. Accepted interventions were further classified into two categories depending on the implementation of the accepted recommendation (“Implemented” or “Not implemented”). Implementation of the recommendation was considered as resolution of the DRPs.

Details of medication-related queries directed to the clinical pharmacist from healthcare staff were recorded on a pre-prepared data collection form.

The attitudes and views of the healthcare staff were obtained from the self-administered questionnaire as described under staff survey above.

### Data analysis

The data were inputted into SPSS, V.21. Sample proportion tests in Minitab 14 were used to calculate the proportions. P values less than 0.05 (*P* < 0.05) were considered to be statistically significant.

## Results

361 and 356 patients were recruited to the intervention and the control arms of the clinical trial, respectively.

### Acceptance of recommendations regarding DRPs

In the intervention arm, a total of 274 DRPs were identified and communicated to the healthcare team (272 to doctors and 2 to nurses) by the clinical pharmacist along with the recommendations for resolving them. Different types of DRPs identified are given in Table 1.

**Table 1:** DRPs communicated to doctors and nurses and their resolution rate

Subcategories of DRPs	Number of DRPs	%	resolved
Doctors (N = 272)			
Unnecessary therapy/No clinical indication	13	92.3%	(12/13)
Untreated indication	162	71.6%	(116/162)
Non reconciled medications	146	72.6%	(106/146)
Inappropriate duration	4	75.0%	(3/4)
Inappropriate dose schedule	11	54.5%	(6/11)
Dose too high	3	33.3%	(1/3)
Dose too low	1	0.0%	(0/1)
Drug-drug interactions	3	100.0%	(3/3)
Inappropriate/inadequate monitoring	1	0.0%	(0/1)
Prescription error	18	100.0%	(18/18)
Manifest side effect, no other cause	7	85.7%	(6/7)
More cost-effective drug available	3	66.7%	(2/3)
Deterioration/improvement of disease state	3	66.7%	(2/3)
Synergistic/preventive drug required and not given	19	63.2%	(12/19)
Duplication of therapy	10	90.0%	(9/10)
Prescribed drug not available	4	75.0%	(3/4)
Avoid contraindications	10	70.0%	(7/10)
Nurses (*N* = 2)			
Charting error	2	100.0%	(2/2)

**Fig. 1:**
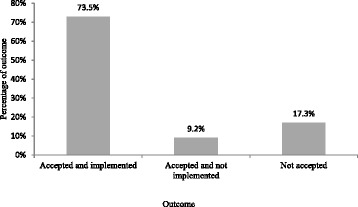
Outcome of pharmacist’s recommendations to doctors

Eighty three percent (225/272); *P* < 0.001 of the recommendations made to the doctors were accepted. 73.5% (200/272); *P* < 0.001) of the recommendations were implemented; i.e. the medical staff made changes to the therapeutic regimen (Fig. 1).

All recommendations on drug-drug interactions and prescription errors were accepted and acted upon (Table 1). A large number of recommendations (162/272) were made to prescribers on untreated indications (i.e. not prescribing a treatment for a particular disease or condition) (Table 1). Of these, the most frequently identified (146/162) were non-reconciliation problems. When there was no documentation or clinically valid reason for not continuing patient’s chronic medications they were described as non-reconciled. A total of 72.6% (106/146) (95% CI 64.6 – 79.7%; *P* < 0.001) of non-reconciled DRPs were rectified after discussion between the prescriber and the clinical pharmacist. Two DRPs about missing the prescribed drugs on the drug chart were identified during data collection and were discussed with nursing staff and resolved.

### Drug information queries directed to the clinical pharmacist

A total of 17 medication-related questions were pro-actively directed to the clinical pharmacist during the seven months study period. Nine were from doctors, 4 were from nurses and four were from medical students. The majority of questions from doctors and medical students were related to the dose and indications of medicines and available generic substitutions in the local market. The nature of the queries from nurses’ was about indication, side effects and generic name of a particular drug (Table 2).

**Table 2:** Medication-related queries directed to the clinical pharmacist

Drug	Query	Directed by
Ferrup (Brand of ferrous sulphate)	What are the dosage forms available in Sri Lanka?	Intern house officers
Folic acid	What are the strengths of the tablets available?	Intern house officers
Tetracycline	What are the possible side effects?	Nurse
Ceftriaxone	What is the best cephalosporin to replace ceftriaxone when therapy is changed to oral administration?	Intern house officers
Ferrous sulphate	What is the usual frequency?	Intern house officers
Rifaximin	What is the indication?	Medical student
Ciprofloxacin	What is the strength of the oral formulation?	Intern house officers
Amifru (Brand of frusemide + amiloride)	What is the generic equivalent?	Intern house officers
Co-trimoxazole	What is the dose?	Intern house officers
Erythromycin	Can this be prescribed for children?	Medical student
Stematil (Prochlrperazine)	What is the generic equivalent?	Nurse
Aspirin	What is the efficacy of enteric coated aspirin in preventing gastric irritation	Medical student
Carticare (Brand of glucosamine)	What is the generic equivalent?	Intern house officers
Paraffin cream	What is the indication?	Nurse
Imuran (Brand of azathioprine)	What is the generic equivalent?	Nurse
Insulin	How to use an insulin pen	Medical student
Tamiflu (Brand of oseltamivir)	What is the generic equivalent?	Intern house officers

### Views and attitudes of the healthcare staff towards clinical pharmacy service

The rate of response of doctors to the post-intervention survey was higher than the rate of response to the baseline survey; 66.7% (8/12) at baseline and 92.3% (12/13) at post-intervention survey. Out of the 8 doctors who participated in the baseline survey 5 completed the post-intervention survey. The survey results showed that all the consultants participated for the baseline and post-intervention survey had worked in UK for their post graduate training.

The views and attitudes of the doctors regarding incorporation of clinical pharmacy service to the existing healthcare system were satisfactory even at the baseline (Additional file 1: Table S1). At the end of the trial intervention period, the majority of doctors (11/12) agreed that the incorporation of a clinical pharmacist to the existing health care system would be useful and this collaboration could improve the current QUM management especially in the public sector hospitals. Ninety two percent (11/12) of doctors would be happy to receive the services from a competent clinical pharmacist. Sixty seven percent (8/12) of doctors accepted that the pharmacist could advise doctors and nurses regarding the issues related to medications. One (8.0%) doctor disagreed with that statement and 25% (3/12) did not express any view. The majority (91.7%) of doctors agreed that adding a pharmacist to the team is not a waste of money and is necessary to the current healthcare system. Sixty seven percent (8/12) of doctors agreed that pharmacists can play an important role in tailoring drug therapy for individual patients. Ninety two percent (11/12) of doctors acknowledged that pharmacist’s contribution is important in improving patients’ medication adherence. The majority of doctors (75.0%) had the opinion that the patients’ awareness about drugs and changes to the patients’ drug therapy was poor.

Doctors views related to the role played by the pharmacist was positive even at the baseline and had improved further following the intervention period (Additional file 3: Table S2). In post-intervention survey more than 75% of doctors agreed that pharmacists can alert the prescriber on adverse drug reactions, interactions and allergies. All the doctors believed that the pharmacist can act as a drug information source to patients and carers. More than 50% of doctors agreed that pharmacists can play an important role in assuring the safety and appropriateness of prescribed medicines.

The response rate of nursing staff was 80.0% (12/15) at the baseline. However the nursing staff did not consent to participate in the post-intervention survey. The perspectives of nursing staff at the baseline were negative; 58.3% (7/12) stated that there is no need of a ward-based clinical pharmacy service and 66.7% (8/12) were not happy to welcome the pharmacy service (Additional file 2: Table S3). The majority of the nursing staff did not agree that a clinical pharmacist could make a useful contribution for patient management (Additional file 4: Table S4).

## Discussion

Clinical pharmacy is a health science discipline which delivers patient-oriented pharmaceutical care, with an aim of improving safe and rational use of medicines [12]. Studies conducted in different parts of the world have shown the benefits of a clinical pharmacy service in terms of reducing negative outcomes of DRPs and improving patient safety [13, 14], improving appropriateness of prescriptions [15, 16] and reducing the impact of healthcare expenditure [17].

In Sri Lankan hospitals clinical pharmacy service does not exist and this is a completely new concept to the existing healthcare environment. This is the first interventional study carried out in Sri Lanka evaluating the acceptance of the recommendations made by a clinical pharmacist by other members of the healthcare team. This study showed that the addition of a clinical pharmacist to the study setting was positively received by the attending doctors. An acceptance rate of 83.0% of pharmacists’ recommendations regarding DRPs by doctors was comparable to developed world settings where acceptance rates range between 63 and 90% [18–20]. The implementation of the pharmacist’s recommendations of 73.5% was comparable to a study done in Sweden by Gillespie, et al. where 75% of the pharmacists’ suggestions were implemented [21]. The acceptance of pharmacist’s recommendations regarding DRPs shows that the physician-pharmacist inter-professional relationship was successfully established in the busy tertiary care hospital where the study was carried out.

The attitudes and views of doctors ranging from specialist physicians, senior registrars and registrars to intern medical officers about the incorporation of clinical pharmacy service and its benefits were positive. A great majority (91.7%) of doctors agreed that adding a pharmacist to the team would be cost effective and is vital to the current healthcare system. Physicians’ perspectives on collaborative work with a clinical pharmacist had been studied widely in many other countries [22–25]. Gillespie, et al. reported that 95% of physicians were satisfied with the incorporation of a clinical pharmacy service to the university hospital in Uppsala in Sweden, which was a new introduction to that setting at that time [22].

However the pharmacist-nurse interaction on team-based patient management had not been explored extensively [22, 26]. In this study, even though a wide proportion of doctors acknowledged the importance of clinical pharmacy service, the response received from the nursing staff was negative as assessed at the baseline. Lack of awareness and knowledge about the importance of clinical pharmacy service in improving QUM and potential benefits to patient care could be the likely reason for this negative response. The survey results showed that none of participated nurses had been worked with a clinical pharmacist in overseas or any private sector hospital. Another survey conducted in Sri Lanka to evaluate the perception of healthcare staff towards the addition of clinical pharmacy service had showed similar findings. According to the results of that survey, 60% of medical staff believed that the incorporation of clinical pharmacists would improve the rational prescribing of medications [27]. However similar to the results of our study the responses of nurses were mostly negative; only 10% appreciated the usefulness of clinical pharmacy service [27]. A study from Pakistan published in 2012, showed a negative perception from nurses towards the role of pharmacist in Pakistan’s healthcare setting [28].

Data from our study emphasizes that there is a need to improve awareness and build trust and relationships with nursing staff in order to demonstrate the potential benefits and promote a clinical pharmacy service in Sri Lankan hospitals.

The foundation for building an efficient team is to encourage learning as a team. Training students on team care at undergraduate and postgraduate levels is likely to improve the awareness of skills and strengths of each category of professionals and help to overcome the barriers for implementation of the clinical pharmacy service [29, 30].

Results of this study demonstrated that the clinical pharmacy service is well accepted and utilized by the medical staff in the tertiary care hospital indicating that there is a significant opportunity for the Sri Lankan clinical pharmacist to make a valuable contribution to enhance the QUM. It also demonstrated that a significant number of DRPs could be identified and acted upon in a manner consistent with best practice multidisciplinary healthcare teams. As in other countries it is reasonable to expect that this will result in improved health outcomes for patients and reduced costs for health system. Thus, our study provides evidence to support health policy change in order to introduce clinical pharmacy service to the healthcare system in Sri Lanka and the other low and middle-income countries (LMICs).

### Strengths and limitations

The study was undertaken in a tertiary level teaching hospital with strong support from lead clinicians. This level of support may not be available in smaller hospitals in the country. Therefore, the results on the acceptance of the pharmacist recommendations by the doctors to resolve DRPs are likely to be generalizable primarily to tertiary hospitals. Tertiary hospitals are a logical place to start as they provide higher exposure of health professionals-in-training to pharmacists being included in multidisciplinary care, which will eventually facilitate expansion to smaller hospitals.

In a study of this duration it is inevitable that there will be turnover of medical staff within the two hospital wards where the study was carried out. The average number of doctors working in both wards was around 12. Thus we were able to offer the survey to a significant proportion of doctors in the clinical team. The numbers of doctors who participated at baseline and after intervention changed mainly because of a change in intern medical officers as they completed their intern period during the study. While this is a limitation in comparison of results it is unlikely that the prior beliefs of the doctors had changed. In addition the continued acceptance of pharmacist recommendations supports the survey observations.

Even though the nurses withdrew from the post-intervention survey, they actually did not withdraw from the clinical trial. As we mentioned the nurses refused to give the consent for participation in the post-intervention staff survey due to a trade union issue. This trade union issue was about the nurses being surveyed and being asked to express their views and not about the pharmacy intervention. Although desirable, within that context it was not possible to perform any kind of in-depth interviews, focus groups or similar qualitative methods with the nursing staff to explore the reasons for their reluctance to collaborate in this component of our study. While the second survey of nursing staff could not be undertaken the 25% of the medication related questions generated by nurses was evidence of nurses acceptance of the trial.

Eventhough all the doctors and nurses participated in the staff survey the number was small. This limits the generalizability of the results generated from the staff survey.

## Conclusions

There was high acceptance of clinical pharmacist’s recommendations regarding DRPs by the other members of the healthcare team and the majority of the DRPs were resolved through this collaboration. The doctors’ views and attitudes were positive regarding the inclusion of a ward-based pharmacist to the healthcare team and they recognized that this collaboration improves QUM in patients. However there is a need to improve liaison between clinical pharmacist and nursing staff.

## Additional files

Additional file 1: Table S1. Attitudes and perceptions of doctors regarding the addition of clinical pharmacists to the healthcare team. (DOCX 14 kb)
Additional file 2: Table S3. Attitudes and perceptions of nurses regarding the addition of clinical pharmacists to the healthcare team. (DOCX 12 kb)
Additional file 3: Table S2. Role of the clinical pharmacist from the doctors’ point of view. (DOCX 12 kb)
Additional file 4: Table S4. Role of the clinical pharmacist from the nurses’ point of view. (DOCX 12 kb)

## Abbreviations

DRPs: Drug related problems
LMICs: Low and middle-income countries
NCCDs: Non-communicable chronic diseases
PCNE: Pharmaceutical Care Network Europe
QUM: Quality use of medicines

## References

[1] Stacey SR, Turner SC, Coulthard KP, Miller H (2013). Paediatric pharmacy in Australia: where have we come from and where do we need to go?. JPPR.

[2] Lozano R, Naghavi M, Foreman K, Lim S, Shibuya K, Aboyans V (2010). Global and regional mortality from 235 causes of death for 20 age groups in 1990 and 2010: a systematic analysis for the global burden of disease study. Lancet.

[3] Murray CJ, Vos T, Lozano R, Naghavi M, Flaxman AD, Michaud C (2010). Disability-adjusted life years (DALYs) for 291 diseases and injuries in 21 regions, 1990–2010: a systematic analysis for the global burden of disease study. Lancet.

[4] Annual health bulletin - Sri Lanka 2012: Medical Statistics Unit, Ministry of Health, Sri Lanka. http://www.health.gov.lk/enWeb/publication/AHB-2012/Annual%20Health%20Bulletin%20-%202012.pdf. Accessed 7 Feb 2015.

[5] Willoch K, Blix HS, Pedersen-Bjergaard AM, Eek AK, Reikvam A (2012). Handling drug-related problems in rehabilitation patients: a randomized study. IJCP.

[6] McAlister FA, Stewart S, Ferrua S, McMurray JV (2004). Multidisciplinary strategies for the management of heart failure patients at high risk for admission: a systematic review of randomized trials. J Am Coll Cardiol.

[7] Satish Kumar BP, Dahal P, Venkataraman R, Fuloria PC. Assessment of clinical pharmacist intervention in tertiary care teaching hospital of southern India. Asian J Pharm Clin Res*.* 2013; 6(Suppl 2).

[8] Chisholm-Burns MA, Zivin JSG, Lee JK, Spivey CA, Slack M, Herrier RN (2010). Economic effects of pharmacists on health outcomes in the United States: a systematic review. AJHP.

[9] Shanika LGT, Jayamanne S,Wijekoon N, Coombes J, Coombes I, Perera D, Mamunuwa N, Pathiraja V, Dawson A, De Silva H A. Usefulness of a ward- based clinical pharmacist in detecting and managing drug related problems: experience from a tertiary care hospital in Sri Lanka. Presented at 74th FIP World Congress of Pharmacy and Pharmaceutical Sciences 2014, Bangkok, Thailand, August 2014 (http://www.postersessiononline.com/173580348_eu/congresos/74fip/aula/-POS-HPS_46_74fip.pdf).

[10] Shanika LGT, Jayamanne S, Wijekoon N, Coombes J, Coombes I, Perera D, Mohamed F, Lynch C, Peters N, Dawson A, De Silva H A. Impact of a ward based clinical pharmacist intervention on improving the quality use of medicines in patients with chronic non-communicable diseases in a tertiary hospital. Presented at 74th FIP World Congress of Pharmacy and Pharmaceutical Sciences 2014, Bangkok, Thailand, August 2014 (http://www.postersessiononline.com/173580348_eu/congresos/74fip/aula/-POS-HPS_45_74fip.pdf).

[11] PCNE Classification for Drug-Related Problems V5.01. www.pcne.org/upload/files/16_PCNE_classification_V5.01.pdf. Accessed 7 Feb 2015.

[12] SHPA (2005). Standards of practice for clinical pharmacy. JPPR.

[13] Scullin C, Scott MG, Hogg A, McElnay JC (2007). An innovative approach to integrated medicines management. J Eval Clin Pract.

[14] Kaboli PJ, Hoth AB, McClimon BJ, Schnipper JL (2006). Clinical pharmacists and inpatient medical care: a systematic review. Arch Intern Med.

[15] Hellström LM, Bondesson Å, Höglund P, Midlöv P, Holmdahl L, Rickhag E (2011). Impact of the Lund integrated medicines management (LIMM) model on medication appropriateness and drug-related hospital revisits. Eur J Clin Pharmacol.

[16] Spinewine A, Swine C, Dhillon S, Lambert P, Nachega JB, Wilmotte L (2007). Effect of a collaborative approach on the quality of prescribing for geriatric inpatients: a randomized, controlled trial. J Am Geriatr Soc.

[17] Gillespie U, Alassaad A, Henrohn D, Garmo H, Hammarlund-Udenaes M, Toss H (2009). A comprehensive pharmacist intervention to reduce morbidity in patients 80 years or older - a randomized controlled trial. Arch Intern Med.

[18] Bergkvist Christensen A, Holmbjer L, Midlöv P, Höglund P, Larsson L, Bondesson Å (2011). The process of identifying, solving and preventing drug related problems in the LIMM-study. Int J Clin Pharm.

[19] Galindo C, Olivé M, Lacasa C, Martínez J, Roure C, Lladó M (2003). Pharmaceutical care: pharmacy involvement in prescribing in an acute-care hospital. Pharm World Sci.

[20] Stemer G, Lemmens-Gruber R (2011). The clinical pharmacist's contributions within the multidisciplinary patient care team of an intern nephrology ward. Int J Clin Pharm.

[21] Gillespie, U. Effects of Clinical Pharmacists' Interventions: on Drug-Related Hospitalisation and Appropriateness of Prescribing in Elderly Patients [PhD thesis]. Faculty of Pharmacy, Uppsala University; 2012. http://www.diva-portal.org/smash/get/diva2:483737/FULLTEXT01.pdf. Accessed 20 Feb 2015.

[22] Gillespie U, Mörlin C, Hammarlund-Udenaes M, Hedström M (2012). Perceived value of ward-based pharmacists from the perspective of physicians and nurses. Int J Clin Pharm.

[23] Farrell B, Pottie K, Woodend K, Yao V, Dolovich L, Kennie N (2010). Shifts in expectations: Evaluating physicians' perceptions as pharmacists become integrated into family practice. J Interprof Care.

[24] Pottie K, Farrell B, Haydt S, Dolovich L, Sellors C, Kennie N (2008). Integrating pharmacists into family practice teams: Physicians’ perspectives on collaborative care. Can Fam Physician.

[25] Dey RM, de Vries MJ, Bosnic-Anticevich S (2011). Collaboration in chronic care: unpacking the relationship of pharmacists and general medical practitioners in primary care. Int J Pharm Pract.

[26] Adamcik BA, Ransford HE, Oppenheimer PR, Brown JF, Eagan PA, Weissman FG (1986). New clinical roles for pharmacists: a study of role expansion. Soc Sci Med.

[27] Mamunuwa AMVGN, Dorabawila SSKBM. The Need for Clinical Pharmacy Services in Sri Lanka; a Study Based on the Prevalence of Drug Related Problems in Two Hospitals. IJSRP. 2014;4(9).

[28] Azhar S, Hassali MA, Ibrahim MIM, Saleem F, Siow-Yen L (2012). A survey evaluating nurses' perception and expectations towards the role of pharmacist in Pakistan's healthcare system. J Adv Nurs.

[29] McPherson K, Headrick L, Moss F (2001). Qual Health Care.

[30] Hughes CM, McCann S (2003). Perceived interprofessional barriers between community pharmacists and general practitioners: a qualitative assessment. Br J Gen Pract.

