# Supplementary material for: Acceptance and attitudes of healthcare staff towards the introduction of clinical pharmacy service: a descriptive cross-sectional study from a tertiary care hospital in Sri Lanka
Source: BMC Health Serv Res. 2017 Jan 18;17:46. doi: 10.1186/s12913-017-2001-1 (PMC5241951; doi:10.1186/s12913-017-2001-1)
Supplement: Additional file 1: Table S1. — Attitudes and perceptions of doctors regarding the addition of clinical pharmacists to the healthcare team. (DOCX 14 kb) [file 12913_2017_2001_MOESM1_ESM.docx]

Additional file 1 Table S1: Attitudes and perceptions of doctors regarding the addition of clinical pharmacists to the healthcare team

|  | **Baseline survey (N = 8)** | | | | | **Post-intervention survey (N = 12)** | | | |  |
| --- | --- | --- | --- | --- | --- | --- | --- | --- | --- | --- |
|  | **SD** | **D** | **N** | **A** | **SA** | **SD** | **D** | **N** | **A** | **SA** |
| Adding a clinical pharmacist to the team is a good idea |  |  |  | 6 | 2 |  |  | 1 | 9 | 2 |
|  |  |  |  | 75.0% | 25.0% |  |  | 8.3% | 75.0% | 16.7% |
| Adding a pharmacist to the team is a waste of money and is completely unnecessary |  | 6  75.0% | 2  25.0% |  |  | 3  25.0% | 8  66.7% | 1  8.3% |  |  |
| Current standard of care in public hospitals in Sri Lanka could be improved by introducing a clinical pharmacist to support the team |  |  |  | 6  75.0% | 2  25.0% |  |  | 1  8.3% | 10  83.4% | 1  8.3% |
| You would be happy to welcome the services of a competent clinical pharmacist to the team |  |  |  | 5  62.5% | 3  37.5% |  |  | 1  8.3% | 10  83.4% | 1  8.3% |
| Patients often do not understand their medicines and the changes that have occurred in hospital |  | 1  12.5% |  | 4  50.0% | 3  37.5% |  | 3  25.0% |  | 6  50.0% | 3  25.0% |
| Errors with medicines happen, but nothing is put in place to prevent these errors happening again | 1  12.5% | 2  25.0% | 1  12.5% | 3  37.5% | 1  12.5% | 1  8.3% | 4  33.3% | 2  16.8% | 4  33.3% | 1  8.3% |
| Pharmacists have no place in advising doctors or nurses about medicine | 1  12.5% | 6  75.0% | 1  12.5% |  |  | 1  8.3% | 7  58.4% | 3  25.0% | 1  8.3% |  |
| Pharmacists can play an important role in tailoring drug therapy for individual patients  patients |  |  | 1  12.5% | 5  63.0% | 2  25.0% |  | 4  33.3% |  | 7  58.4% | 1  8.3% |
| Pharmacists can play an important role in improving patient adherence to medication regimens |  |  |  | 6  75.0% | 2  25.0% | 1  8.3% |  |  | 9  75.0% | 2  16.7% |

SD = Strongly Disagree/ D = Disagree/ N = No opinion/ A = Agree/ SA = Strongly Agree
