# Supplementary material for: Acceptance and attitudes of healthcare staff towards the introduction of clinical pharmacy service: a descriptive cross-sectional study from a tertiary care hospital in Sri Lanka
Source: BMC Health Serv Res. 2017 Jan 18;17:46. doi: 10.1186/s12913-017-2001-1 (PMC5241951; doi:10.1186/s12913-017-2001-1)
Supplement: Additional file 2: Table S3. — Attitudes and perceptions of nurses regarding the addition of clinical pharmacists to the healthcare team. (DOCX 12 kb) [file 12913_2017_2001_MOESM2_ESM.docx]

Additional file 3: Table S2: Role of the clinical pharmacist from the doctors’ point of view

| **The role of the clinical pharmacists** | **Baseline survey**  **(N = 8)**  **(n=8)** | | **End survey**  **(N = 12)**  **(n=12)** | |
| --- | --- | --- | --- | --- |
|  | **Yes** | **No** | **Yes** | **No** |
| Checking whether patient allergies are documented | 4  (50%) | 4  (50%) | 10  (83%) | 2  (17%) |
| Alerting the prescriber to any drug interactions that may have been overlooked | 6  (75%) | 2  (25%) | 10  (83%) | 2  (17%) |
| Alerting the prescriber to any suspected adverse drug reactions – documenting and providing assistance with management where necessary | 5  (63%) | 3  (37%) | 9  (75%) | 3  (25%) |
| Teaching patient or carer on administration | 6  (75%) | 2  (25%) | 12  (100%) | 0  (00%) |
| Ensuring all drugs and doses are safe and appropriate for a patient | 5  (63%) | 3  (37%) | 8  (67%) | 4  (33%) |
| Assisting staff with administration techniques of medicines (eg. Noting before or after food, how fast to administer an IV antibiotic) | 5  (63%) | 3  (37%) | 10  (83%) | 2  (17%) |
| Annotating the drug chart with any tips to minimize drug error (eg. writing the generic name of the drug when the brand has been prescribed) | 5  (63%) | 3  (37%) | 9  (75%) | 3  (25%) |
| Ensuring that all changes to a patients therapy are intentional and not due to unintentional error | 4  (50%) | 4  (50%) | 7  (58%) | 5  (42%) |
