# Supplementary material for: Acceptance and attitudes of healthcare staff towards the introduction of clinical pharmacy service: a descriptive cross-sectional study from a tertiary care hospital in Sri Lanka
Source: BMC Health Serv Res. 2017 Jan 18;17:46. doi: 10.1186/s12913-017-2001-1 (PMC5241951; doi:10.1186/s12913-017-2001-1)
Supplement: Additional file 3: Table S2. — Role of the clinical pharmacist from the doctors’ point of view. (DOCX 12 kb) [file 12913_2017_2001_MOESM3_ESM.docx]

Additional file 2: Table S3: Attitudes and perceptions of nurses regarding the addition of clinical pharmacists to the healthcare team

|  | **Baseline survey (N = 12)** | | | | |
| --- | --- | --- | --- | --- | --- |
|  | **SD** | **D** | **N** | **A** | **SA** |
| Adding a clinical pharmacist to the team is a good idea | 3  25.0% | 4  33.0% | 3  25.0% | 1  8.0% | 1  8.0% |
| Adding a pharmacist to the team is a waste of money and is completely unnecessary | 0  0.0% | 2  17.0% | 4  33.0% | 5  42.0% | 1  8.0% |
| Current standard of care in public hospitals in Sri Lanka could be improved by introducing a clinical pharmacist to support the team | 1  8.0% | 4  33.0% | 4  33.0% | 3  25.0% | 0  0.0% |
| You would be happy to welcome the services of a competent clinical pharmacist to the team | 4  33.0% | 4  33.0% | 2  17.0% | 2  17.0% | 0  0.0% |
| Patients often do not understand their medicines and the changes that have occurred in hospital | 0  0.0% | 3  25.0% | 3  25.0% | 6  50.0% | 0  0.0% |
| Errors with medicines happen, but nothing is put in place to prevent these errors happening again | 1  8.0% | 5  42.0% | 5  42.0% | 1  8.0% | 0  0.0% |
| Pharmacists have no place in advising doctors or nurses about medicine | 1  8.0% | 4  33.0% | 2  17.0% | 4  33.0% | 1  8.0% |
| Pharmacists can play an important role in tailoring drug therapy for individual patients | 1  8.0% | 6  50% | 4  33.0% | 1  8.0% | 0  0.0% |
| Pharmacists can play an important role in improving patient adherence to medication regimens | 1  8.0% | 3  25.0% | 5  42.0% | 3  25.0% | 0  0.0% |

SD = Strongly Disagree/ D = Disagree/ N = No opinion/ A = Agree/ SA = Strongly Agree
