# Supplementary material for: Acceptance and attitudes of healthcare staff towards the introduction of clinical pharmacy service: a descriptive cross-sectional study from a tertiary care hospital in Sri Lanka
Source: BMC Health Serv Res. 2017 Jan 18;17:46. doi: 10.1186/s12913-017-2001-1 (PMC5241951; doi:10.1186/s12913-017-2001-1)
Supplement: Additional file 4: Table S4. — Role of the clinical pharmacist from the nurses’ point of view. (DOCX 12 kb) [file 12913_2017_2001_MOESM4_ESM.docx]

| **The role of the clinical pharmacists** | **Baseline survey (N = 12)** | |
| --- | --- | --- |
|  | **Yes** | **No** |
| Checking whether patient allergies are documented | 2  (17%) | 10  (83%) |
| Alerting the prescriber to any suspected adverse drug reactions – documenting and providing assistance with management where necessary | 0  (0%) | 12  (100%) |
| Alerting the prescriber to any drug interactions that may have been overlooked | 0  (0%) | 12  (100%) |
| Teaching patient or carer on administration | 2  (17%) | 10  (83%) |
| Ensuring all drugs and doses are safe and appropriate for a patient | 2  (17%) | 10  (83%) |
| Assisting staff with administration techniques of medicines (eg. Noting before or after food, how fast to administer an IV antibiotic) | 1  (8%) | 11  (92%) |
| Annotating the drug chart with any tips to minimize drug error (eg. writing the generic name of the drug when the brand has been prescribed) | 1  (8%) | 11  (92%) |
| Ensuring that all changes to a patients therapy are intentional and not due to unintentional error | 0  (0%) | 12  (100%) |

Additional file 4: Table S4: Role of the clinical pharmacist from the nurses’ point of view
